# *Enhancer of zeste* plays an important role in photoperiodic modulation of locomotor rhythm in the cricket, *Gryllus bimaculatus*

**DOI:** 10.1186/s40851-016-0042-7

**Published:** 2016-03-19

**Authors:** Yoshimasa Hamada, Atsushi Tokuoka, Tetsuya Bando, Hideyo Ohuchi, Kenji Tomioka

**Affiliations:** Graduate School of Natural Science and Technology, Okayama University, 3-1-1 Tsushima-naka, Kita-ku, Okayama 700-8530 Japan; Graduate School of Medicine, Dentistry and Pharmaceutical Sciences, Okayama University, Kita-ku, Okayama 700-8558 Japan

**Keywords:** Circadian rhythm, Photoperiodic modulation, Histone modification, Clock gene, Enhancer of zeste

## Abstract

**Introduction:**

Insects show daily behavioral rhythms controlled by an endogenous oscillator, the circadian clock. The rhythm synchronizes to daily light–dark cycles (LD) and changes waveform in association with seasonal change in photoperiod.

**Results:**

To explore the molecular basis of the photoperiod-dependent changes in circadian locomotor rhythm, we investigated the role of a chromatin modifier, *Enhancer of zeste* (*Gb*’*E*(*z*)), in the cricket, *Gryllus bimaculatus*. Under a 12 h:12 h LD (LD 12:12), *Gb*’*E*(*z*) was constitutively expressed in the optic lobe, the site of the biological clock; active phase (α) and rest phase (ρ) were approximately 12 h in duration, and α/ρ ratio was approximately 1.0. When transferred to LD 20:4, the α/ρ ratio decreased significantly, and the *Gb*’*E*(*z*) expression level was significantly reduced at 6 h and 10 h after light-on, as was reflected in the reduced level of trimethylation of histone H3 lysine 27. This change was associated with change in clock gene expression profiles. The photoperiod-dependent changes in α/ρ ratio and clock gene expression profiles were prevented by knocking down *Gb*’*E*(*z*) by RNAi.

**Conclusions:**

These results suggest that histone modification by *Gb*’*E*(*z*) is involved in photoperiodic modulation of the *G. bimaculatus* circadian rhythm.

## Introduction

Most animals exhibit daily rhythms in various physiological functions that synchronize with daily environmental cycles, such as light–dark cycles (LD), which are affected by Earth’s rotation [1]. The rhythm is generated by a circadian clock, which is an endogenous mechanism that oscillates over a period of approximately 24 h. The circadian clock’s oscillatory mechanism is based on transcriptional/translational molecular feedback loops [2–4]. In insects, the clock machinery has been studied most extensively in the fruit fly, *Drosophila melanogaster*, in which the major players are *Clock* (*Clk*) and *cycle* (*cyc*) [2, 3]; their product proteins, CLK and CYC, form heterodimers and activate the transcription of *period* (*per*) and *timeless* (*tim*) during the late day to early night. The translated proteins PER and TIM accumulate in the cytoplasm during the night, and in late night they heterodimerize and enter the nucleus to repress their own transcription by inhibiting CLK-CYC transcriptional activity. This feedback results in a reduction of PER and TIM levels, leading to the reactivation of *per* and *tim* transcription [2, 3].

Recent studies have revealed that circadian clock cycling is precisely controlled by mechanisms that include chromatin remodeling, recruitment of RNA polymerases, and post-transcriptional and post-translational modifications [5–8]. Chromatin remodeling plays an important role in regulating the circadian clock and in its response to environmental time cues. In both mammals and insects, CLK acts as a transcriptional activator and recruits other transcription factors by binding to E-boxes at the regulatory regions of clock-controlled genes, including *per* and *tim* [9–11].

In addition to daily time-keeping, the circadian clock plays a key role in seasonal changes in physiology, including the change in active phase to rest phase ratio (α/ρ ratio), based on seasonal change in photoperiod [12–14]. The photoperiod-dependent change in α/ρ ratio persists for many days in constant darkness and is recognized as a kind of history-dependent change in the circadian clock. History-dependent changes are also observed in the free-running period [15–17]. However, the molecular basis of photoperiod-dependent changes has remained elusive.

In the present study, we investigated the possible involvement of chromatin modification in photoperiod-dependent changes in circadian rhythm of the cricket, *Gryllus bimaculatus*. We used the cricket in this study for the following reasons: first, the cricket shows a clear photoperiodic response in locomotor activity rhythms [12, 14]; second, the circadian clock has been localized in the optic lobe [18, 19]; and third, cDNAs were previously obtained for major clock genes [20]. Although number of chromatin modifiers so far known for insects including E(z), UTX, TRX, and Su(var)3-9 [21–24], we investigated the role of E(z) in photoperiodic modulation of the circadian rhythm, since its mammalian homolog EZH2 has been shown to be involved in control of the circadian clock mechanism through trimethylation of histone H3 lysine 27 (H3K27) [25] and we have some experimental evidence that RNA interference (RNAi) of *E*(*z*) effectively reduces H3K27 trimethylation (H3K27me3) level in our cricket [24]. We found that knockdown of *E*(*z*) expression by RNAi prevented photoperiodic modulation. The results are discussed relative to the role of E(z) in photoperiodic modulation as well as seasonal adaptation of the circadian clock.

## Materials and methods

### Animals

Adult male crickets were purchased or taken from our laboratory colony, which is maintained under standard conditions of LD 12:12, at a constant temperature of 25 °C. The crickets were fed laboratory chow and water.

### Measurement of mRNA levels

Quantitative real-time RT-PCR (qPCR) was used to measure mRNA levels. Total RNA was extracted from optic lobes of 4–6 adult males using ISOGEN (Nippon Gene, Japan) or TRIzol Reagent (Invitrogen, California). The total RNA was treated with DNase I to remove any traces of genomic DNA. Approximately 250 ng of total RNA of each sample was reverse transcribed with random 6mers using PrimeScript RT Reagent Kit (Takara, Japan). qPCR was performed with an Mx3000P Real-time PCR System (Stratagene, California) using Fast Start Universal SYBR Green Master (Roche, Japan) including SYBR Green with the following primers for respective genes: 5′-AAGGTGCGAAAACAGGCATC-3′ and 5′-TCGTCGTTTTGGTGGATGTG-3′ for *Gb*’*E*(*z*) (GenBank Accession No. LC012934), 5′-AAGCAAGCAAGCATCCTCAT-3′ and 5′-CTGAGAAAGGAGGCCACAAG-3′ for *Gb*’*per* (GenBank Accession No. AB375516), 5′-GATTATGAAGTCTGTGATGATTGG-3′ and 5′-AGCATTGGAGAGAACTGAA-GAGGT-3′ for *Gb*’*tim* (GenBank Accession No. AB548625), 5′-GGCCGAAGCTCATAAAGTGG-3′ and 5′-AACCGCACAAAGGAACCATC-3′ for *Gb*’*cyc* (GenBank Accession No. AB762416), 5′-AATGACCGTAGTCGAGAAAGTGAAG-3′ and 5′-TTGCGATGATTGAGGTTGTTG-3′ for *Gb*’*Clk* (GenBank Accession No. AB738083), and 5′-GCTCCGGATTACATCGTTGC-3′ and 5′-GCCAAATGCCGAAGTTCTTG-3′ for *Gb*’*rpl18a* (GenBank Accession No. DC448653). Standard curves for the transcripts were generated by serial dilutions of amplified cDNAs and included in each qPCR run. After 40 PCR cycles, samples were subjected to melting curve analysis, and a single expected amplicon was confirmed in each sample. The results were analyzed using software associated with the instrument: quantification of mRNA levels was performed by the standard curve method, and the values were normalized to the values of *rpl18a* at each time point.

### RNAi

Double-stranded RNAs (dsRNAs) were synthesized using the MEGAScript T7 Kit (Ambion, California) and adjusted to 20 μM for *DsRed2* and 2 μM for *Gb*’*E*(*z*) for RNAi. For dsRNA synthesis, we used the T7 primer, 5′-TAATACGACTCACTATAGGG-3′, for *Gb*’*E*(*z*) and an exogenous gene, *DsRed2*; dsRNA lengths were 431 bp and 660 bp, respectively. In total, 700 nL of dsRNA was injected into the abdomens of the adult male crickets. As a negative control, we injected dsRNA for *DsRed2*.

### Immunohistochemistry

Heads of adult males were fixed at *zeitgeber* time (ZT) 10 (ZT 0 corresponds to lights-on) with 2 % paraformaldehyde (PFA) in phosphate-buffered saline with 0.1 % Tween 20 (PBT) for 17 h at 4 °C. The brain and optic lobe complex was extracted using a fine razor knife, tweezers, and micro scissors under dissecting microscope, then fat bodies and associated nerves were removed. The tissues were refixed in 4 % PFA/PBT and dehydrated in 25 %, 50 %, 75 % EtOH/PBT and 100 % EtOH. Dehydrated samples were rehydrated in 75 %, 50 %, and 25 % EtOH/PBT, washed with PBT, and blocked with 1 % normal goat serum (NGS) in PBT for 1 h. Blocked samples were incubated overnight at 4 °C with rabbit polyclonal anti-trimethylated H3K27 antibody (EMD Millipore, Germany; 1:500 diluted in 1 % NGS in PBT). The samples were then washed with PBT and incubated for 3 h with secondary anti-rabbit IgG antibody conjugated with Alexa Fluor 488 (Molecular Probes, Massachusetts; 1:500 diluted in 1 % NGS/PBT). Samples were washed with PBT and incubated with DAPI at 1:1000 in PBT for 15 min. The samples were mounted on a glass slide with 50 % glycerol/PBT, observed and optical digital images were captured with a confocal laser scanning microscope (LSM-780, Zeiss, Germany).

To quantify the staining intensity of H3K27me3 and DAPI, the fluorescence intensities were quantified from digital images of six optic lobes for each treatment using the ImageJ software (freely available at http://rsb.info.nih.gov/ij/). The H3K27me3 staining indices were shown as the ratio of staining intensity of H3K27me3 to that of DAPI.

### Recording of locomotor activity

Locomotor activity of individual animals was recorded with an actograph made of a transparent plastic box (18 × 9 × 4.5 cm) with a rocking substratum, as previously described [26]. A magnetic reed switch sensed rocking movements of the substratum caused by a moving cricket. The number of movements was recorded every 6 min by a computerized system. Water and food were provided ad libitum. The activity chambers were placed in an incubator in which temperature was kept at 25 ± 0.5 °C and desired lighting regimens were provided by a cool white fluorescent lamp connected to an electric timer. The light intensity was 600–1000 lux, which varied based on the animal’s proximity to the lamp.

The raw data were displayed as conventional double-plotted actograms to judge activity patterns, and free-running periods were calculated using the *χ*^2^ periodogram [27] in ActogramJ [28]. If a peak of the periodogram was above the 5 % confidence threshold, the peak period was designated as statistically significant. The duration of the active phase, or subjective night, (α) was estimated with ActogramJ: the boundary of the active phase was defined at the time point where the moving average of activity exceeded or fell below 70 % of daily average activity; then, a linear regression line was fitted to the points for consecutive days. The rest of the time was designated as the rest phase, or subjective day, (ρ).

### Statistical analysis

The one-way analysis of variance (ANOVA) followed by a post hoc Tukey-test was used to compare the differences in means of free-running periods, α/ρ ratios, and immunostaining intensity of H3K27me3 between the control and experimental groups. Significance of daily cycling of clock gene mRNA levels and difference of mRNA levels among three groups of crickets treated in different ways were tested by ANOVA followed by Tukey-test. To compare the means of two groups, *t*-test was used. In all statistical tests, the significance level was set at *P* < 0.05.

## Results

### Expression profile of *Gb*’*E*(*z*) in the optic lobe and its suppression by RNAi

**Fig. 1:**
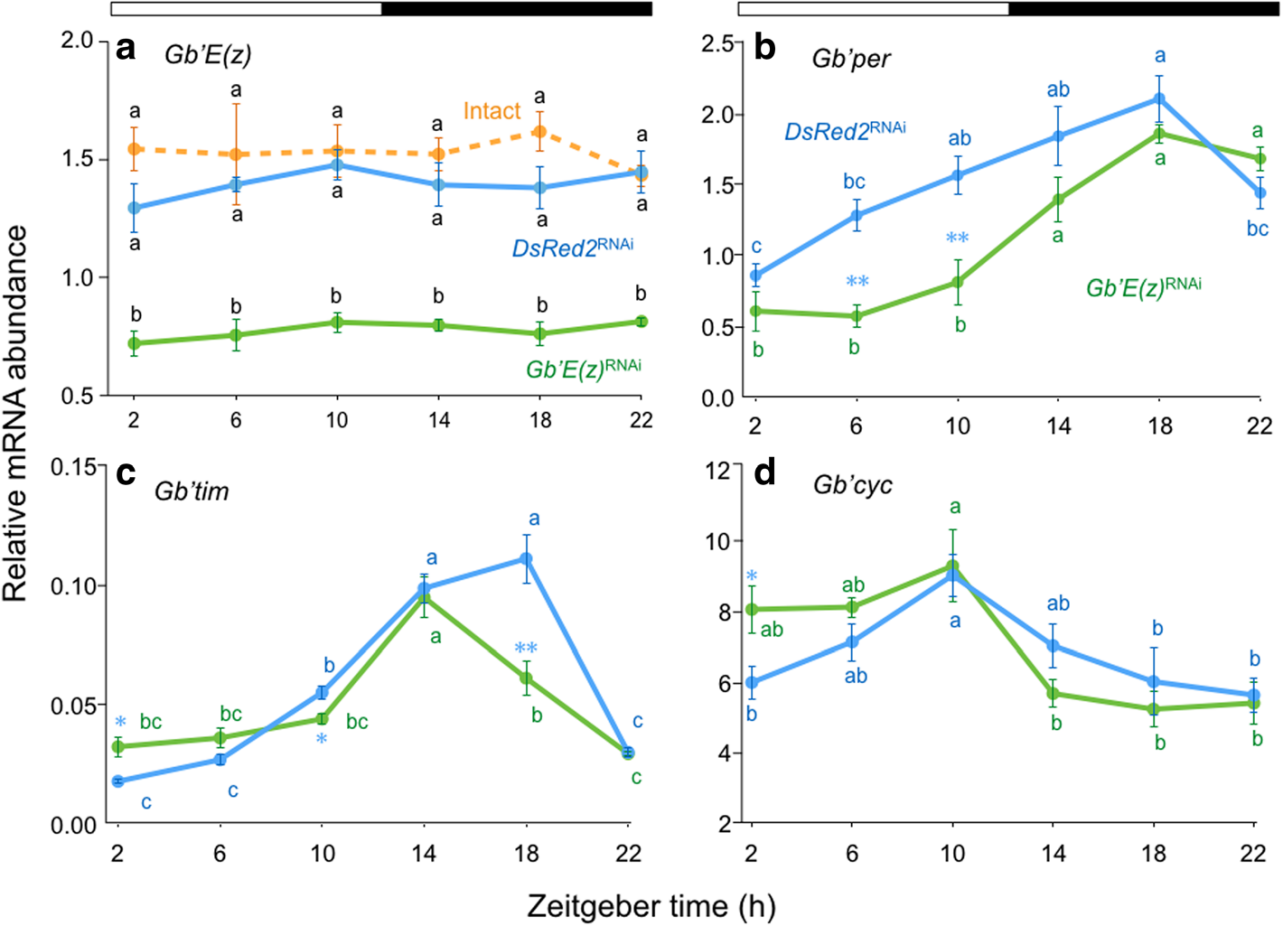
Daily expression profiles of *Gb*’*E*(*z*) and clock genes in the optic lobe of the cricket *Gryllus bimaculatus* under LD 12:12. Blue and green symbols with solid lines indicate the mRNA levels of *Gb*’*E*(*z*) (**a**), *Gb*’*per* (**b**), *Gb*’*tim* (**c**), and *Gb*’*cyc* (**d**) in *DsRed2* ^RNAi^ and *Gb*’*E*(*z*)^RNAi^ crickets, respectively. For *Gb*’*E*(*z*) data (**a**), intact crickets are also shown by orange symbols. In RNAi crickets, the optic lobes were collected seven days after dsRNA injection. mRNA abundance was measured by qPCR, with total RNA extracted from the optic lobes. Data collected from 3–15 independent measurements were averaged and plotted as mean ± SEM. The values shown are relative to those of *Gb*’*rpl18a* mRNA, which was used as an internal reference. For *Gb*’*E*(*z*), values with different lower case letters at each ZT significantly differ from each other (Tukey-test, *P* < 0.01). For *Gb*’*per*, *Gb*’*tim*, and *Gb*’*cyc*, *P < 0.05, **P < 0.01, *t*-test vs *DsRed2* ^RNAi^ and values with different lower case letters of the same color differ significantly from each other (Tukey-test, *P* < 0.05). The *Gb*’*E*(*z*) mRNA showed no daily rhythms in intact, *DsRed2* ^RNAi^, or *Gb*’*E*(*z*)^RNAi^ crickets, whereas the mRNA of other three genes showed a clear oscillation in both *DsRed2* ^RNAi^ and *Gb*’*E*(*z*)^RNAi^ crickets (ANOVA, *P* < 0.01)

We first examined whether *Gb*’*E*(*z*) was expressed in the optic lobe, which is known to harbor the circadian clock in the cricket, by using qPCR to measure *Gb*’*E*(*z*) mRNA levels in the tissue. Under LD 12:12 *Gb*’*E*(*z*) mRNA was fairly consistently expressed throughout a day in intact crickets (Fig. 1a), and no daily fluctuation was observed (ANOVA, F_5, 41_ = 0.34, *P* > 0.88).

To estimate the effects of *Gb*’*E*(*z*)^RNAi^, we measured *Gb*’*E*(*z*) levels in the optic lobe of *Gb*’*E*(*z*)^RNAi^ crickets and control (*DsRed2*^RNAi^) crickets, which were treated with ds*DsRed2. DsRed2*^RNAi^ crickets showed similar *Gb*’*E*(*z*) expression to that of intact crickets (Fig. 1a) and no daily rhythm was observed (ANOVA, F_5, 37_ = 0.52, *P* > 0.76). The value at each time point did not differ from that of intact crickets (Tukey-test, *P* > 0.05). In *Gb*’*E*(*z*)^RNAi^ crickets, *Gb*’*E*(*z*) expression was greatly reduced compared with the control and intact crickets at all time points (Tukey-test, *P* < 0.01). Expression reduced to approximately 53 % of that of intact and control crickets at ZT 10. These results indicate that *Gb*’*E*(*z*) is expressed rather constitutively in the optic lobe and could be knocked down by RNAi against *Gb*’*E*(*z*), and that *DsRed2*^RNAi^ crickets can be used as a control.

### Expression profile of clock genes in the optic lobe under LD 12:12

Expression profiles of the clock genes *Gb*’*per*, *Gb*’*tim*, and *Gb*’*cyc* were investigated in the optic lobe of *DsRed2*^RNAi^ and *Gb*’*E*(*z*)^RNAi^ crickets under LD 12:12 by qPCR. The results are shown in Fig. 1b–d. In the control crickets, the mRNA levels of *Gb*’*per* and *Gb*’*tim* showed a significant daily fluctuation, which peaked at ZT 18 under LD 12:12 (ANOVA: F_5, 36_ = 9.01, *P* < 0.01 for *Gb*’*per*, F_5, 17_ = 67.97, *P* < 0.01 for *Gb*’*tim*) (Fig. 1b, c). *Gb*’*cyc* also showed a significant daily cycling, with a peak at ZT 10 (ANOVA: F_5, 41_ = 3.62, *P* < 0.01) (Fig. 1d). Similar rhythmic expression profiles were observed for the three genes in *Gb*’*E*(*z*)^RNAi^ crickets (ANOVA: F_5, 20_ = 15.47, *P* < 0.01 for *Gb*’*per*; F_5, 17_ = 24.25, *P* < 0.01 for *Gb*’*tim*; F_5, 22_ = 4.45, *P* < 0.01 for *Gb*’*cyc*), but with slight changes in pattern or phase compared with those in *DsRed2*^RNAi^ crickets (Fig. 1b-d): *Gb*’*per* showed a significant reduction at ZT 6 and ZT 10 (*t*-test, *P* < 0.01), and *Gb*’*tim* showed a slight increase at ZT 2, a slight reduction at ZT 10 (*t*-test, *P* < 0.05), and a reduction at ZT 18 (*t*-test, *P* < 0.01) that resulted in a phase advance of the peak by 4 h. Moreover, *Gb*’*cyc* showed a significant increase at ZT 2 (*t*-test, *P* < 0.05).

### Effects of *Gb*’*E*(*z*)^RNAi^ on circadian locomotor rhythms in LD 12:12 and ensuing DD

**Fig. 2:**
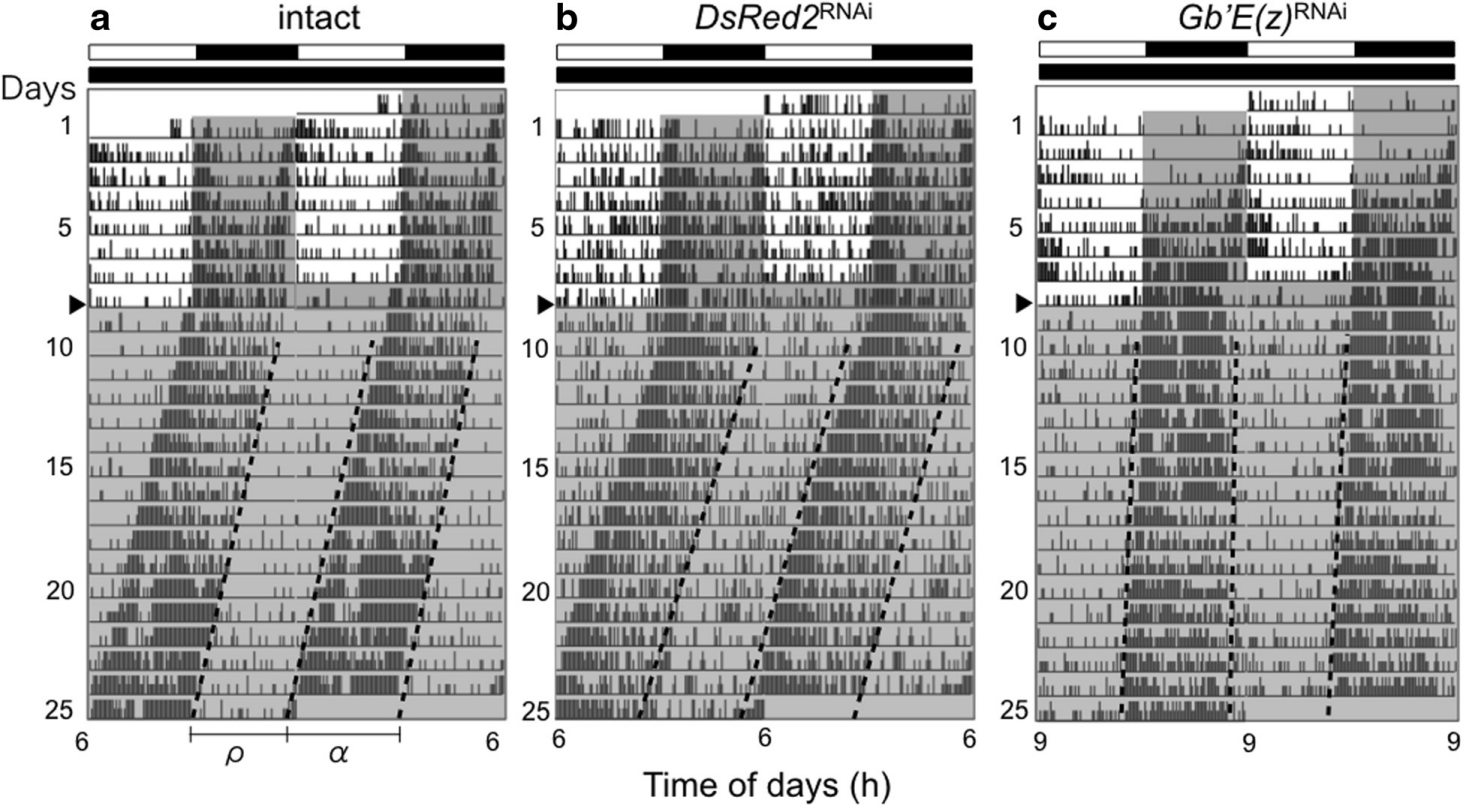
Double-plotted actograms of locomotor activity of adult male crickets *Gryllus bimaculatus* under LD 12:12 and DD. **a**, **b**, and **c** show representative records of intact crickets (**a**) and those treated with dsRNA against *DsRed2* (**b**) or *Gb*’*E*(*z*) (**c**). Arrowheads indicate the day when the crickets were transferred from LD 12:12 to DD, at a constant temperature of 25 ± 0.5 °C. α and ρ indicate active phase and rest phase, respectively. Dotted lines indicate the onset and offset of the active phase. White and black bars above the actogram indicate the light and the dark phase, respectively. Gray area in the actogram indicates the dark phase

To examine the role of *Gb*’*E*(*z*) in regulation of circadian rhythms, we recorded the locomotor activity in *Gb*’*E*(*z*)^RNAi^, intact and control crickets. Both intact and control crickets showed nocturnal activity rhythms that peaked just after lights-off under LD 12:12, and the rhythm persisted with a free-running period slightly shorter than 24 h in constant darkness (DD) (Fig. 2a, b, Table 1). We measured the duration of active phase (α) and rest phase (ρ), and calculated α/ρ ratio to characterize the daily activity profile. The α/ρ ratios of intact and control crickets were approximately 1.0 both in LD 12:12 and DD (Table 1). These results were consistent with those of our previous study [14]. The *Gb*’*E*(*z*)^RNAi^ crickets showed nocturnal locomotor rhythms similar to those of control crickets under LD 12:12 (Fig. 2c); their α/ρ ratios were approximately 1.0 both in LD 12:12 and DD (Table 1), and were not significantly different from those of the intact and control crickets (ANOVA followed by Tukey-test, *P* > 0.05). However, their average free-running period was significantly longer than that of intact and control crickets (ANOVA followed by Tukey-test, *P* < 0.01, Table 1), which suggests that methylation of H3K27 is involved in regulating the free-running period in DD.

**Table 1:** Effects of RNAi against *Gb*’*E*(*z*) and *DsRed2* on the locomotor rhythm of *Gryllus bimaculatus*

Treatment	n	Light Condition	τ (mean ± SD) h	α/ρ ratio (mean ± SD)
LD12:12				
intact	13	LD	-	0.94 ± 0.07^a^
	12	DD	23.66 ± 0.25^a^	0.95 ± 0.23^a^
*DsRed2* ^RNAi^	23	LD	-	0.97 ± 0.09^a^
	18	DD	23.63 ± 0.33^a^	0.97 ± 0.11^a^
*Gb’E*(*z*)^RNAi^	26	LD	-	0.99 ± 0.08^a^
	23	DD	23.97 ± 0.16^b^	1.02 ± 0.11^a^
LD20:4				
intact	23	LD	-	0.53 ± 0.20^cd^
	13	DD	23.80 ± 0.12^ab^	0.46 ± 0.20^d^
*DsRed2* ^RNAi^	33	LD	-	0.69 ± 0.22^bc^
	22	DD	23.70 ± 0.23^a^	0.55 ± 0.19^cd^
*Gb’E*(*z*)^RNAi^	23	LD	-	0.84 ± 0.28^ab^
	17	DD	23.99 ± 0.16^b^	0.83 ± 0.25^ab^

ANOVA revealed significant difference in both free-running period (τ) (F_5, 98_ = 9.01, *P* < 0.01) and α/ρ ratio (F_11, 230_ = 22.0, *P* < 0.01). Values with different lower case letters significantly differ from each other (Tukey-test, *P* < 0.01, except for α/ρ ratios between intact in DD following LD 20:4 and *DsRed2* ^RNAi^ in LD 20:4, where *P* < 0.05)

### Role of *Gb*’*E*(*z*) in photoperiodic modulation of locomotor rhythms

**Fig. 3:**
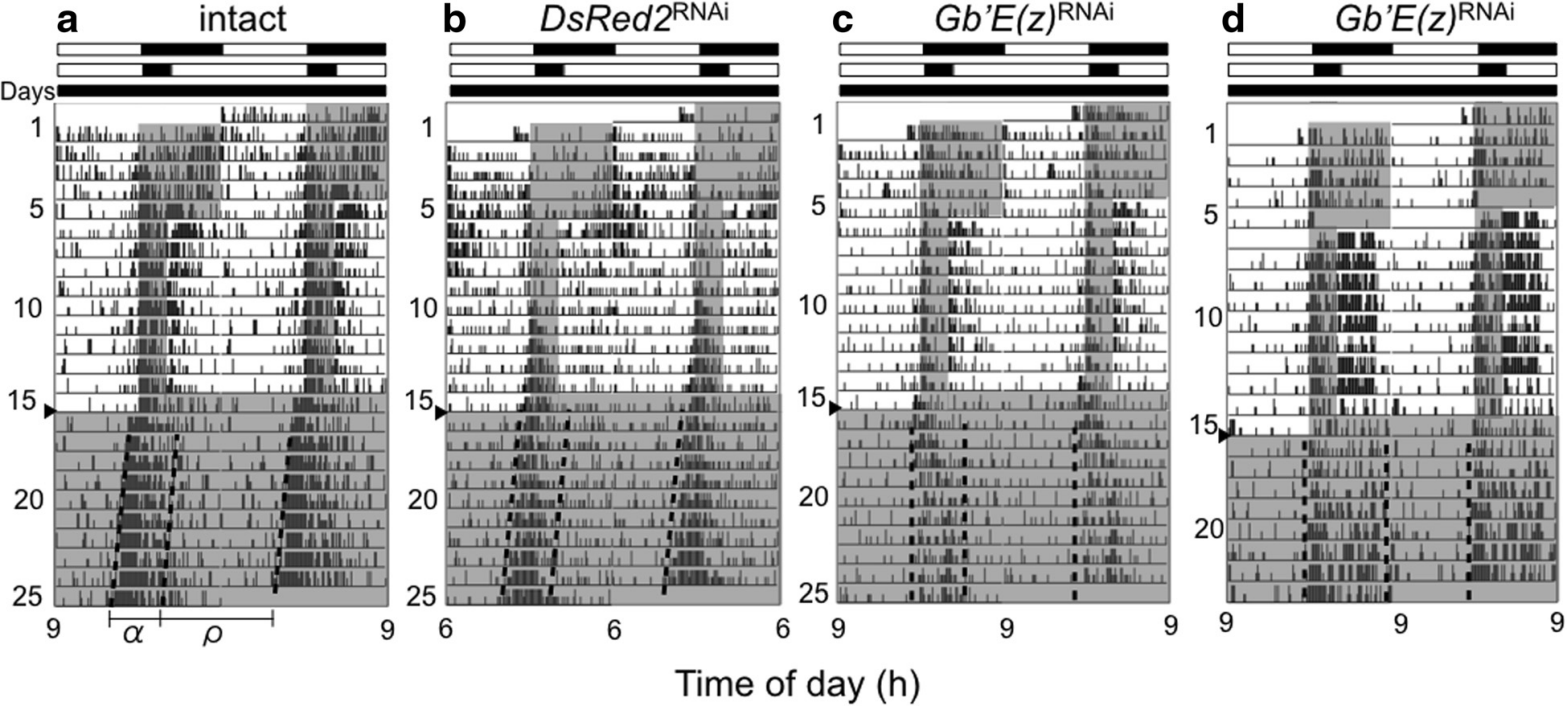
Double-plotted actograms of locomotor activity of adult male crickets *Gryllus bimaculatus* under LD 12:12, LD 20:4, and DD. (**a**) intact cricket, (**b**) cricket treated with ds*DsRed2*, (**c**, **d**) crickets treated with ds*Gb*’*E*(*z*). Crickets were exposed to LD 12:12 for the first 5 days, then in LD 20:4 for 9 days, and transferred to DD on the day indicated by an arrowhead, at a constant temperature of 25 ± 0.5 °C. α and ρ indicate active phase and rest phase, respectively. Dotted lines indicate onset and offset of the active phase. White and black bars above the actogram indicate the light and dark phases, respectively. Gray area in the actogram indicates the dark phase

In the cricket, the α/ρ ratio in DD has been shown to change depending on the preceding photoperiodic conditions [14]. We used LD 20:4 to examine the photoperiodic modulation of the rhythm because it has been shown to reduce the α/ρ ratio most effectively [14]. To examine the effects of *Gb*’*E*(*z*)^RNAi^ on this photoperiodic effect on the α/ρ ratio, we recorded locomotor activity rhythm of intact, *DsRed2*^RNAi^, and *Gb*’*E*(*z*)^RNAi^ crickets under LD 12:12, followed by LD 20:4 for 9 cycles, and then by DD. The representative records are shown in Fig. 3, and the locomotor rhythm parameters are summarized in Table 1. The intact and control crickets showed that locomotor activity was confined to a short dark phase under LD 20:4, and the shortened active phase persisted under subsequent free-running conditions (Fig. 3a, b).

The α/ρ ratios of intact and *DsRed2*^RNAi^ crickets under LD 20:4 and the ensuing DD were both significantly smaller than those in LD 12:12 and the ensuing DD (Table 1). There was no significant difference in the α/ρ ratio between intact and *DsRed2*^RNAi^ crickets under DD (ANOVA followed by Tukey-test, *P* > 0.05). These results are consistent with those of Koga et al. [14]. Under LD 20:4 some *Gb*’*E*(*z*)^RNAi^ crickets (6/23) showed similar changes in α/ρ ratio (Fig. 3c). However, the remaining crickets (17/23) exhibited an activity pattern that consisted of a strong activity bout after lights-on in addition to the light-off peak under LD 20:4; the average α/ρ ratio was 0.84 ± 0.28 (*n* = 23) (Table 1). Interestingly, the α/ρ ratio was maintained in the ensuing DD, and was significantly greater than those of the intact and control crickets (ANOVA followed by Tukey-test, *P* < 0.01, Table 1). The free-running period of the *Gb*’*E*(*z*)^RNAi^ crickets was again significantly longer than that of the control crickets (Tukey-test, *P* < 0.01). These results suggest that *Gb*’*E*(*z*) is involved in photoperiodic modulation and regulation of free-running period in the circadian locomotor rhythm.

### Involvement of *Gb*’*E*(*z*) in photoperiodic modulation of clock gene expression profiles

**Fig. 4:**
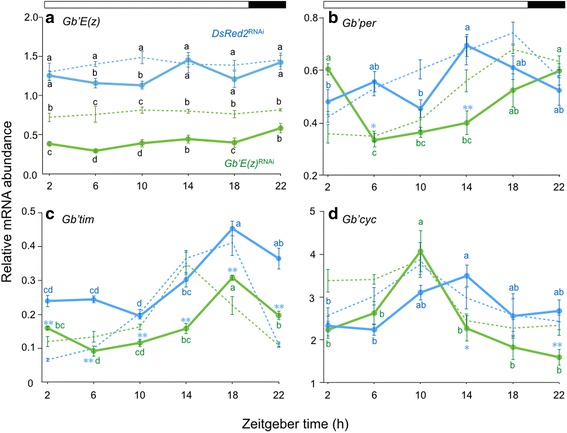
Daily expression profiles of *Gb*’*E*(*z*) and clock genes in the optic lobe of the cricket *Gryllus bimaculatus* under LD 20:4. Blue and green symbols with solid lines indicate the mRNA levels of *Gb*’*E*(*z*) (**a**), *Gb*’*per* (**b**), *Gb*’*tim* (**c**), and *Gb*’*cyc* (**d**) in *DsRed2* ^RNAi^ and *Gb*’*E*(*z*)^RNAi^ crickets, respectively. For reference, data for LD 12:12 are shown by broken lines (blue, *DsRed2* ^RNAi^ crickets; green, *Gb*’*E*(*z*)^RNAi^ crickets). mRNA abundance was measured by qPCR, with total RNA extracted from the optic lobes collected seven days after transfer to LD 20:4. Data collected from 3–10 independent measurements were averaged and plotted as mean ± SEM. The values shown are relative to those of *Gb*’*rpl18a* mRNA, which was used as an internal reference. In LD 20:4, *Gb*’*E*(*z*) mRNA showed no significant daily oscillation in *DsRed2* ^RNAi^ crickets (ANOVA, *P* > 0.05) but a significantly lower level at ZT 6 and 10 in comparison with that in LD 12:12 (**a**). *Gb*’*E*(*z*)^RNAi^ reduced the *Gb*’*E*(*z*) levels which were even lower than those of *Gb*’*E*(*z*)^RNAi^ crickets in LD 12:12 at ZT 2–10 and ZT 18 (Tukey-test, *P* < 0.01). The values with different lower case letters at each ZT significantly differ from each other (Tukey-test, *P* < 0.01, except between *DsRed2* ^RNAi^ in LD 20:4 and *Gb*’*E*(*z*)^RNAi^ in LD 12:12 at ZT 10 and ZT 18 where *P* < 0.05). The mRNA levels of *Gb*’*per* (**b**), *Gb*’*tim* (**c**), and *Gb*’*cyc* (**d**) showed clear oscillatory profiles in both *DsRed2* ^RNAi^ and *Gb*’*E*(*z*)^RNAi^ crickets (ANOVA, *P* < 0.05 for *Gb*’*cyc* in *DsRed2* ^RNAi^, and *P* < 0.01 for all other combinations). Values with different lower case letters differ significantly from each other (Tukey-test, *P* < 0.05). *P < 0.05, **P < 0.01, *t*-test vs *DsRed2* ^RNAi^ crickets in LD 20:4

Because *Gb*’*E*(*z*) is the chromatin modifier that controls gene expression through H3K27 methylation, we predicted that *Gb*’*E*(*z*) is involved in photoperiodic modulation of α/ρ ratio through alteration in clock gene expression. Consequently, we examined whether endogenous *Gb*’*E*(*z*) mRNA levels in the optic lobe altered with a change of photoperiodic condition (Fig. 4a). In the control crickets, *Gb*’*E*(*z*) showed no significant daily change in its expression, but a significant reduction was found at ZT 6 and ZT 10 in LD 20:4 when compared with that in LD 12:12 (Tukey-test, *P* < 0.01). *Gb*’*E*(*z*)^RNAi^ highly reduced *Gb*’*E*(*z*) levels compared with the control at all the points in LD 20:4 (Tukey-test, *P* < 0.01). The reduced levels were significantly less at ZT 2–10 and ZT 18 than those with same treatment under LD 12:12 (Tukey-test, *P* < 0.01 for ZT 2–10 and *P* < 0.05 for ZT 18). These results indicate that *Gb*’*E*(*z*) expression level is affected by photoperiod and may be involved in photoperiodic modulation of locomotor rhythm.

We then examined the expression profiles of the clock genes *Gb*’*per*, *Gb*’*tim*, and *Gb*’*cyc* in the optic lobe in the control and *Gb*’*E*(*z*)^RNAi^ crickets in LD 20:4 by qPCR (Fig. 4b–d). In the control crickets, *Gb*’*per* and *Gb*’*cyc* mRNA levels showed significant daily fluctuation (ANOVA: F_5, 26_ = 3.97, *P* < 0.01 for *Gb*’*per*; F_5, 27_ = 3.48, *P* < 0.05 for *Gb*’*cyc*). The peak phase occurred during the late day phase (ZT 14), which indicates that *Gb*’*per* and *Gb*’*cyc* oscillations advanced and delayed by approximately 4 h, respectively, compared with those in the preceding LD 12:12 (Fig. 4b, d). *Gb*’*tim* mRNA levels also showed significant daily cycling, with a peak at ZT 18 (ANOVA: F_5, 26_ = 17.50, *P* < 0.01), retaining the phase relationship to the lights-on similar to that was seen in LD 12:12 (Fig. 4c). In *Gb*’*E*(*z*)^RNAi^ crickets, mRNA levels of the three clock genes showed significant daily fluctuation (ANOVA: F_5, 27_ = 12.09, *P* < 0.01 for *Gb*’*per*; F_5, 18_ = 37.93, *P* < 0.01 for *Gb*’*tim*; F_5, 38_ = 7.58, *P* < 0.01 for *Gb*’*cy*c). *Gb*’*tim* showed a phase relationship with LD 20:4 similar to that in the control crickets, but the mRNA levels were significantly reduced (*t*-test, *P* < 0.01). Interestingly, the peak phase of *Gb*’*per* and *Gb*’*cyc* was delayed and advanced by approximately 8 and 4 h, respectively, relative to control crickets (Fig. 4b, d). These results suggest that expression profiles of *Gb*’*per* and *Gb*’*cyc* were altered by photoperiod and that this alteration was mediated at least in part by *Gb*’*E*(*z*).

### Methylation levels of histone H3K27

**Fig. 5:**
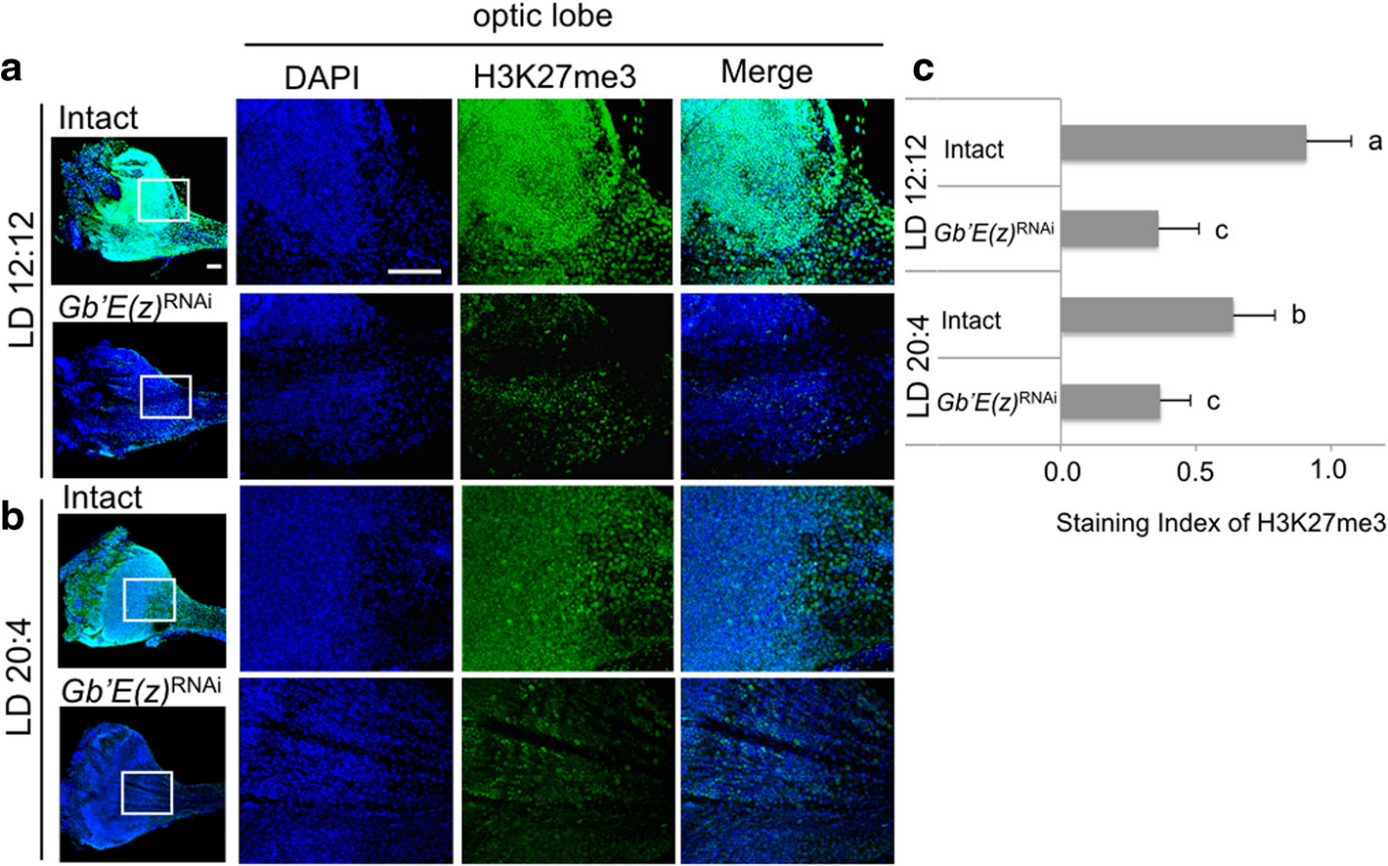
Levels of histone H3 trimethylated at K27 (H3K27me3) in the optic lobe of the cricket *Gryllus bimaculatus*. (**a**, **b**) location of H3K27 in the optic lobes under LD 12:12 (**a**) or LD 20:4 (**b**). The right three columns indicate high magnification images of the optic lobe indicated by square from the low-magnification image in the left column. All images are oriented with dorsal side at the top. Scale bar, 100 μm. Blue and green indicate nuclei (DAPI) and H3K27me3, respectively. (**c**) Staining indices of H3K27me3 in intact and *Gb*’*E*(*z*)^RNAi^ crickets in LD 12:12 and LD 20:4. Average of 6 optic lobes are shown with SD. Values with different lower case letters differ significantly from each other (ANOVA followed by Tukey-test, *P* < 0.05). Note that level of H3K27me3 is higher in LD 12:12 than LD 20:4 and is greatly reduced in *Gb*’*E*(*z*)^RNAi^ crickets

Since expression levels of *Gb*’*E*(*z*) were lower in LD 20:4 than in LD 12:12, we examined the level of histone H3 methylated at K27 at ZT 10 in the two photoperiodic conditions by immunohistochemistry using anti-H3K27me3 antibody. In both conditions, many nuclei were labeled by the antibody showing trimethylation of H3K27, being distributed over whole optic lobe (Fig. 5a, b). The expression levels in the optic lobe were significantly higher in LD 12:12 than in LD 20:4 (Fig. 5c). In the crickets treated with *Gb*’*E*(*z*)^RNAi^, the levels of H3K27me3 in the optic lobe were significantly reduced both in LD 12:12 and LD 20:4 (Fig. 5). These results suggest that *Gb*’*E*(*z*) contributes to the modulation of histone H3K27me3 levels in the optic lobe. However, no significant difference was found between *Gb*’*E*(*z*)^RNAi^ crickets of the two photoperiodic conditions (Fig. 5c), although the *Gb*’*E*(*z*) mRNA levels significantly differed (Fig. 4a). This may be explained that the *Gb*’*E*(*z*) protein levels were so reduced in both cases that no significant difference was detected in H3K27me3 levels.

## Discussion

Using an RNAi knockdown approach, we showed that the chromatin modifier *Gb*’*E*(*z*) is involved in regulating the free-running period and photoperiodic modulation of the locomotor rhythm in the cricket, *G. bimaculatus*.

### *Gb*’*E*(*z*) expression in the optic lobe

Our qPCR analysis revealed that the chromatin modifier *Gb*’*E*(*z*) is constitutively expressed in the optic lobe, which is the location of the cricket’s circadian clock [19], which recalls the fact that most of the chromatin modifiers involved in circadian gene expression are present on a constitutive basis and recruited as needed to regulate transcription [7]. In mammals, for example, CLOCK plays a key role in the event and forms a transcriptional complex with BMAL1 to activate *Per* and *Cry* transcription by binding to the E-box in their upstream region [29]. Meanwhile, CLOCK acts as a factor that opens up chromatin, which enables other transcriptional factors to act on target genes of CLOCK [11]. Similar molecular events may occur in transcription of *per* and *tim* in *Drosophila*, as CLK-CYC binding to upstream and/or intronic E-boxes of *per* and *tim* controls chromatin modifications through H3K9 acetylation and H3K4 trimethylation [10].

Interestingly, however, *Gb*’*E*(*z*) mRNA levels at ZT 6 and ZT 10 were higher in LD 12:12 than LD 20:4 (Fig. 4a) and the difference was reflected in the trimethylation levels of H3K27 (Fig. 5). To our knowledge, this is the first evidence for photoperiodic regulation of a clock-related chromatin modifier in insects. Light-induced modification of chromatin is known for H3S10 in the mammalian circadian clock in the suprachiasmatic nucleus [30]; it is a result of phosphorylation and occurs within 5 min after lights-on, which is similar to *c*-*fos* induction. *Gb*’*E*(*z*) seems to be regulated by a different mechanism than immediate induction of phosphorylation, because no apparent change was observed at lights-on in *Gb*’*E*(*z*) mRNA levels (Figs. 1a and 4a). KISMET, a chromatin-remodeling enzyme, represents a candidate regulator for this photoperiodic regulation of *Gb*’*E*(*z*), as it is required for normal circadian light responses in *Drosophila* [31]. *Gb*’*E*(*z*) was also shown to be induced by mechanical stimulation; when a leg was injured, the onset of regeneration was associated with upregulation of *Gb*’*E*(*z*) [24, 32]. Thus, its expression seems to be regulated by multiple pathways.

### *Gb*’*E*(*z*) may be involved in photoperiodic modulation of the circadian rhythm

Involvement of chromatin modifiers in regulating the circadian clock has been shown in animals. In *Drosophila*, Brahma chromatin-remodeling protein regulates CLK binding to target promoters, and hence the free-running period of the rhythm [8]. Mice that lacked *Mettle3*, a factor that regulates RNA methylation, showed a delay in RNA processing that leads to circadian period elongation [33]. However, our results revealed that *Gb*’*E*(*z*) plays a different role from those previously described regulatory models. *Gb*’*E*(*z*) knockdown significantly lengthened the free-running period in DD after LD 12:12 and LD 20:4 (Table 1). Another important effect of *Gb*’*E*(*z*)^RNAi^ was elimination of the photoperiod-dependent change in α/ρ ratio. In control crickets, the active phase was usually compressed during the dark phase in LD 20:4; consequently, the α/ρ ratio became much smaller (0.69 ± 0.22) than that in LD 12:12 (0.97 ± 0.09) (Table 1). The α/ρ ratio further decreased when transferred to DD (0.55 ± 0.19) (Table 1). These results are consistent with our previous results [14]. However, compression of the active phase was only observed in a few *Gb*’*E*(*z*)^RNAi^ crickets, and the majority of them showed no compression; the average α/ρ ratio was 0.84 ± 0.28 and 0.83 ± 0.25 in LD 20:4 and in the ensuing DD, respectively (Table 1). These results strongly suggest that regulation by E(z) is required in the response to a changing photoperiod through modulating the duration of the active phase. The link between regulatory mechanisms and circadian waveform or free-running period should be investigated in future studies.

### *Gb*’*E*(*z*) may contribute to response to photoperiodic changes via clock genes

This study revealed that temporal expression profiles of clock genes change in response to LD cycle. Under a long-day condition of LD 20:4, the circadian expression profile of the clock gene *Gb*’*tim* showed a similar pattern to that in LD 12:12, whereas *Gb*’*per* and *Gb*’*cyc* expression was advanced and delayed by 4 h, respectively (Fig. 4). *Gb*’*E*(*z*)^RNAi^ prevented the shift of *Gb*’*per* and *Gb*’*cyc* rhythms in response to LD 20:4 (Fig. 4), which suggests the involvement of *Gb*’*E*(*z*) in photoperiod-dependent phase setting of daily clock gene expression. This function of *Gb*’*E*(*z*) may be mediated by trimethylation of histone H3K27 because H3K27me3 levels are dependent on *Gb*’*E*(*z*) mRNA levels (Fig. 5) and H3K27me3 is known to regulate expression of *Per1* and *Per2* in the mammalian circadian clock [25]. In the mammalian clock, EZH2, a homologous protein of E(z), is recruited by the CLOCK/BMAL1 complex to bind to H3 in the promoter regions of *Per1* and *Per2* and causes di- and trimethylation of H3K27 [25]. The phase shift of *Gb*’*cyc* cycling caused by *Gb*’*E*(*z*)^RNAi^ might be attributable to indirect effects through the change of *Gb*’*per* rhythm, because *Gb*’*per* is involved in the transcriptional regulation of *Gb*’*cyc* [34].

The photoperiodic regulation of daily clock gene expression may be caused by a change in *Gb*’*E*(*z*) mRNA levels (Fig. 4a). Because no daily rhythm is known in binding ability of EZH2 to CLOCK: BMAL1 and H3 at the promoter regions of *Per1* and *Per2* [25], quantity of E(z) may affect the binding and eventually daily expression profiles of the clock genes. The change in *Gb*’*E*(*z*) levels may not be a simple response to a given photoperiod, because the α/ρ ratio established in a photoperiod is maintained for a long period in DD [14]. The mechanism of this long-lasting change is an important issue that should be addressed in future studies.

The long-lasting response to photoperiod is reminiscent of photoperiodism regulating the seasonal physiological adaptation in insects. Many cricket species also show photoperiodic responses [35]. For example, nymphal development of *Modicogryllus siamensis* is photoperiodically regulated; nymphs grow faster under long-day conditions and become adults after seven moltings, whereas under short-day conditions, the nymphal period is elongated and the number of molts to adulthood increases [36]. Interestingly, developmental time course is determined within about 10 days after hatching [36], which indicates a long-lasting effect of photoperiod. This is consistent with the finding in this study suggesting that the long-lasting change in the clock is caused by an epigenetic mechanism, and photoperiodic response is most likely underlain by the circadian clock [37, 38]. In addition to the histone modification, we also have to consider the role of DNA methylation, because a recent study showed that maternal transfer of photoperiodic information to offspring is attributable to DNA methylation in *Nasonia vitripennis* [39].

The means by which changes in molecular oscillation are reflected in overt activity rhythms are still being elucidated. There are lines of evidence that molecular oscillation of the circadian clock changes in a photoperiod-dependent manner. In *Drosophila*, daily *per* expression, i.e., phase and ratio of splicing variants, changes in response to photoperiod [40–42]. In other insects, the expression profiles of clock genes also reportedly changed based on photoperiod [43, 44]. In the present study, we showed that *Gb*’*per* and *Gb*’*cyc* responded differently so that the peak phases of their daily expression rhythms became closer in the long photoperiod (Fig. 4). Although additional studies are necessary, this phase change might be somehow related to the shortening of the active phase under long-day conditions.

## Conclusion

The present study discovered for the first time that methylation on H3K27 by *Gb*’*E*(*z*) is required for photoperiodic modulation of the circadian locomotor rhythm, such as length of the active phase and free-running period, which is associated with changes in expression profiles of clock component genes. These results contribute to molecular dissection of the mechanisms of insect photoperiodism and photoperiodic modulation in circadian rhythms.
